# Single-Stage Versus 2-Stage Facial Reanimation With a Free Functional Muscle Flap: Protocol for a Systematic Review

**DOI:** 10.2196/64009

**Published:** 2025-08-21

**Authors:** Oluwatobi Adegboye, Muzammil Arif Din, Jodika Gilworth, Foiz Ahmed, Manaf Khatib

**Affiliations:** 1 Faculty of Health, Education, Medicine, and Social Care Anglia Ruskin University Chelmsford United Kingdom; 2 University of Cambridge Cambridge United Kingdom; 3 Education & Research Centre Wythenshawe Hospital Manchester United Kingdom; 4 Department of Plastic Surgery East and North Hertfordshire NHS Trust Stevenage United Kingdom

**Keywords:** facial palsy, facial reanimation, plastic surgery, single-stage technique, 2-stage technique

## Abstract

**Background:**

Facial paralysis is a condition that has several etiologies and is associated with significant physical and psychosocial complications. Classically, 2-stage facial reanimation was used to treat patients with unilateral facial paralysis; however, single-stage facial reanimation has seen an increase in use. Studies comparing both techniques are limited, and to the best of our knowledge, no systematic reviews have compared these techniques.

**Objective:**

We aim to perform a systematic review that explores how the outcomes of single-stage facial reanimation with a free functional muscle flap compare against those of 2-stage facial reanimation.

**Methods:**

This systematic review protocol has been written according to the PRISMA-P (Preferred Reporting Items for Systematic Review and Meta-Analysis Protocols) and NICE (National Institute for Health and Care Excellence) guidelines. The search strategy will involve 3 stages: electronic databases (MEDLINE, Embase, and Web of Science Core Collection), trial registers (ClinicalTrials.gov), and snowballing. Studies published from 1995 to 2025 will be screened using inclusion and exclusion criteria. Data will be extracted into a standardized spreadsheet, and the risk of bias and quality of evidence will be assessed. Two reviewers will screen, extract, and assess the included studies. Discrepancies will be discussed and rectified with a third reviewer, if needed. A narrative synthesis of the findings will be performed and presented with descriptive and statistical analyses, where possible, using GraphPad Prism (GraphPad Software).

**Results:**

The review formally began in February 2025 and will be performed and reported according to this protocol and the PRISMA (Preferred Reporting Items for Systematic Reviews and Meta-Analyses) guidelines. A PRISMA flow diagram and checklist have been used to summarize the current search and study selection. A narrative synthesis and tables will be used to summarize the results of the included studies. Where possible, statistical analyses will be presented using graphs and figures. The final systematic review is expected to be published in December 2025.

**Conclusions:**

This systematic review will aim to summarize the outcomes of the single-stage and 2-stage facial reanimation techniques and explore the differences between these surgical techniques, if any. The results will provide updated information for patients and clinicians. Moreover, the review may help identify potential areas for further research and policy development.

**Trial Registration:**

PROSPERO CRD42024556255; https://www.crd.york.ac.uk/PROSPERO/view/CRD42024556255

**International Registered Report Identifier (IRRID):**

PRR1-10.2196/64009

## Introduction

The loss of function associated with facial muscle movement is termed facial paralysis or facial nerve palsy. Facial nerve damage arising from various etiologies can cause facial paralysis. The most common cause of facial paralysis is Bell's palsy, an acute idiopathic unilateral lower motor facial nerve lesion [1]. Other common causes of facial paralysis are cerebrovascular events, facial trauma, viral infections (notably herpes zoster), bacterial infections, neoplasms, and congenital causes, including birth trauma and syndromic conditions such as Moebius syndrome. Bilateral facial paralysis is rare, often caused by systemic manifestations of a disease, and it occurs in up to 2% of all cases [2]. Facial paralysis is associated with a poor quality of life, likely because of physical and psychosocial sequelae. Altered facial appearance, difficulty with swallowing and speech, and ophthalmic complications may occur [3-6]. Patients with facial paralysis may experience altered self-perception, social anxiety, and depression [7]. The etiology and onset of facial paralysis determine the treatment. In acute-onset Bell's palsy, the urgent administration of high-dose corticosteroids is indicated [8]. Other medical adjuncts for acute facial palsy include antivirals, such as acyclovir, especially if the facial palsy is associated with herpes zoster infection (Ramsay Hunt syndrome). Surgical intervention is indicated if medical therapy is unsuccessful or facial paralysis has been present for many months [9]. Within a year of onset, nerve reconstruction to stimulate native muscles may be effective for patients. In facial palsy lasting over a year, there is irreversible fibrosis of the neuromuscular junction, and therefore, nerve reconstructive surgery is unlikely to lead to improved facial nerve function [10].

Facial reanimation surgery using free functional muscle flaps, such as the gracilis muscle flap, provides smile potential in facial paralysis. These free muscle flaps have been reported to provide effective long-term outcomes even in long-standing facial paralysis [11-13]. Since its conception in the 1970s, 2-stage facial reanimation using free muscle flaps has been widely accepted in the surgical management of long-standing unilateral facial palsy [14,15]. The first stage of this approach involves establishing a cross-facial nerve graft (CFNG) by connecting the sural nerve to the proximal facial nerve on the unaffected (contralateral) side. The second stage involves a separate operation for free muscle transfer and coaptation to the CFNG [16]. This is the single innervation technique. In the dual innervation technique, a CFNG and nerve to the masseter are used to restore facial muscle function. The immediate drawbacks of 2-stage facial reanimation are the need for 2 separate operations and the risk of complications secondary to nerve grafting, such as paraesthesia.

Single-stage facial reanimation was developed in 1998, with researchers reporting faster reinnervation times and impressive cosmetic outcomes [17,18]. In single-stage facial reanimation, the nerve of the free muscle flap may be connected to the contralateral facial nerve using a CFNG, connected to the ipsilateral nerve to the masseter (a branch of the trigeminal facial nerve), or connected to both (dual innervation) within a single operation. This avoids multiple operations, and if a CFNG is not used, this approach avoids nerve grafting. Recent studies have shown the key benefits of single-stage facial reanimation, namely stronger smile excursion and faster muscle neurotization and recovery [18-20]. Textbox 1 summarizes the benefits and risks of the single-stage and 2-stage facial reanimation techniques according to the current literature.

**Benefits and risks of single-stage and 2-stage facial reanimation using a free functional muscle flap.**

**Single-stage facial reanimation**

***Benefits***

- Single recovery
- Lower anesthetic risk

***Risks***

- Increased complexity
- Further operations may still be needed

**Two-stage facial reanimation**

***Benefits***

- More planned approach
- More predictable outcome

***Risks***

- Increased surgical or anesthetic time
- Increased recovery time

There are limited randomized controlled trials and systematic reviews reporting on facial reanimation surgery [21-35]. Most studies discuss the optimal types of free muscle flaps, nerve reconstructive techniques, or patient-reported outcome measures (PROMs). There is a paucity in the literature of comparisons between the single-stage and 2-stage reanimation techniques, with only 1 systematic review published a few years ago [30]. Understanding the broad outcomes and patient experiences of these surgical techniques is important to provide patients with up-to-date and accurate information when consenting to facial reanimation. Moreover, it is vital for clinicians to better understand the indications, effectiveness, and complications of the single-stage and 2-stage facial reanimation techniques. Consequently, this proposed systematic review aims to explore how the outcomes of single-stage facial reanimation compare against those of 2-stage facial reanimation. Ideally, the results of this review will further inform clinical practice and provide collated evidence for future research and guidelines.

## Methods

### Protocol Registration

This systematic review protocol was registered with the International Prospective Register of Systematic Reviews (PROSPERO), a database produced by the Centre of Reviews and Dissemination and funded by the National Institute for Health Research (NIHR). The registration information is as follows: PROSPERO 2024 CRD42024556255 [36]. This review protocol has been reported with guidance from the PRISMA-P (Preferred Reporting Items for Systematic Review and Meta-Analysis Protocols) and NICE (National Institute for Health and Care Excellence) checklists [37,38]. The systematic review, once completed, will be reported according to the PRISMA (Preferred Reporting Items for Systematic Reviews and Meta-Analyses) guidelines [39].

### Study Question

This study will explore how the outcomes of single-stage facial reanimation with a free functional muscle flap compare against those of 2-stage facial reanimation. This will be in the context of patients with unilateral facial palsy undergoing surgical facial reanimation to correct their condition.

### Search Strategy

A qualified librarian (JG) has devised the search strategy with support from the lead author (OA) as a subject specialist. The question has been broken into concepts using PICO (population, intervention, comparison, and outcomes) (Table 1) and not according to outcome because this review strategy has been suggested to introduce additional biases, such as outcome reporting bias [40]. The strategy has been peer reviewed by librarian colleagues at a tertiary National Health Service hospital trust and approved by the lead author before being adapted individually to each database and trial registry.

**Table 1:** PICO (population, intervention, comparison, and outcomes) framework.

Characteristic	Information
Patient	Patients who underwent facial reanimation surgery for unilateral facial palsy
Intervention	Single-stage facial reanimation using a free functional muscle flap
Comparison	Two-stage facial reanimation using a free functional muscle flap
Main outcome	Clinician-reported outcomes of facial symmetry
Additional outcomes	Patient-reported outcome measures Adverse outcomes

The main outcomes include clinician-reported outcomes of facial symmetry, such as smile excursion, recovery time for facial movements, and nerve regeneration rate. The first additional outcome subcategory includes PROMs such as the FACE-Q instrument [41,42]. The second additional outcome subcategory includes adverse outcomes such as operative complications.

The search will be carried out in 3 stages. The first stage will investigate published studies in the listed electronic bibliography databases. MEDLINE, Embase, and Web of Science Core Collection will be searched because of their medical and surgical content. The second stage will investigate trial registers, such as ClinicalTrials.gov (via Cochrane Library), to identify ongoing or recently completed trials to screen for eligible studies with published data that may be included. The third and final stage will involve searching the reference lists of eligible primary studies (“backward snowballing”) as well as studies that have cited these primary studies (“forward snowballing”) [43]. Example search strategies for the first search stage (MEDLINE via Ovid) and the second search stage (ClinicalTrials.gov via Cochrane Library) are displayed in Multimedia Appendices 1 and 2, respectively.

Only human studies written in the English language and published between 1995 and 2025 will be included. The search will not be limited by country of origin; however, studies published in languages other than English will be excluded. Studies including animals will also be excluded. Unpublished studies, dissertations, conference proceedings, and grey literature identified during the search will be excluded.

The search strategy will be rerun before the final analysis to identify further eligible studies so that the most contemporary literature will be presented in the review. We will aim to include the full search strategies for all databases in the published systematic review.

### Study Selection and Data Extraction

Study screening and selection will be performed according to the PICO framework and exclusion criteria. The exclusion criteria for this review will be studies involving patients with facial palsy due to trauma alone and patients with bilateral facial palsy. Traumatic facial palsy often differs in outcomes because of associated injuries to the head, neck, and other regions of the body [44]. Moreover, additional management options for facial nerve palsy, such as facial nerve decompression, may affect the use of free functional muscle flaps [45]. Bilateral facial palsy was excluded mainly due to the surgical approach. Often, there are preferred muscle flaps or nerves used in bilateral facial palsy, which may affect patient recovery and outcomes compared with unilateral palsy [10,46-49].

Studies that include single-stage or 2-stage facial reanimation or compare both will be included. Textbox 2 displays the types of studies that will be included and excluded in the review. Quasirandomized trials are trials that have the same approach as randomized controlled trials but use a method of allocation that is not truly random [50].

**Types of studies included and excluded.**

**Types of studies included**

- Randomized controlled trials or quasirandomized trials
- Cohort studies
- Case series (with at least 10 patients included)
- Mixed methods studies (quantitative and qualitative methods used)

**Types of studies excluded**

- Reviews (literature or systematic review, with or without meta-analysis)
- Case-control studies
- Case reports and case series (with less than 10 patients included)
- Qualitative-only studies

Rayyan software will be used to screen and select eligible studies [51]. Initially, 1 reviewer (OA) will screen the titles and abstracts of all studies identified by the search strategy against the eligibility criteria. The full text of studies that pass the initial stage of screening will be retrieved and independently screened once more by the same reviewer. Afterwards, a second reviewer (MK) will independently examine the screening process. Disagreements will be settled by discussion between the 2 reviewers; however, if deemed necessary, a third reviewer (FA) will be involved.

Data will be extracted from the included studies using the Systematic Review Repository Plus software and stored in a secure, standardized database [52]. Textbox 3 displays which data will be extracted in the review.

One reviewer will independently extract data (OA), and a second reviewer will evaluate the extracted data (MK). Disagreements will be settled by discussion between the 2 reviewers; however, if deemed necessary, a third reviewer (FA) will be involved. Missing data from the included studies will be requested from the authors via email. Missing data will be extracted and analyzed using the same methodology as that used for the previously gathered datasets.

**Data extraction strategy.**

**Study setting**

- Full citation
- Year
- Country
- Study design
- Methodology
- Study duration
- Length of follow-up

**Patient demographics**

- Number of patients
- Age
- Sex
- Etiology of facial palsy
- Baseline clinical status including severity
- Side of facial palsy

**Intervention details**

- Surgical intervention or technique
- Operating time
- Details of the free flap
- Donor site morbidity
- Innervation details
- Recovery time

**Comparator details**

- Presence of a comparator group
- Surgical intervention or technique
- Operating time
- Details of the free flap
- Donor site morbidity
- Innervation details
- Recovery time

**Outcome measures**

- Clinician-reported outcomes of facial symmetry
- Patient-reported outcome measures
- Adverse outcomes

### Risk of Bias and Quality of Evidence

The risk of bias of the included studies will be assessed with consideration of the criteria recommended by the International Cochrane Collaboration [53]. This includes randomization sequence generation (only valid for true and quasirandomized controlled trials), treatment allocation concealment, blinding, completeness of outcome data, selective outcome reporting, and other sources of bias. The ROBINS-I (Risk of Bias in Nonrandomized Studies-of Interventions) tool will be used to assess the risk of bias in nonrandomized studies included in this review, such as quasirandomized trials and cohort studies [54]. The RoB 2 (revised risk of bias tool for randomized trials) tool will be used to assess the risk of bias in randomized controlled trials [55]. The included studies will also be assessed for quality of evidence using the GRADE (Grading of Recommendations, Assessment, Development, and Evaluations) framework [56,57]. Each study will be graded from very low to high (GRADE certainty ratings) on each of the GRADE domains, and the study’s overall quality will be based on the lowest scoring domain. Two reviewers (OA and JG) will independently assess the risk of bias and quality of evidence of the included studies. Disagreements will be settled by discussion between the 2 reviewers; however, if deemed necessary, a third reviewer (MK or FA) will be involved. Tables will be used to display the risk of bias and quality of evidence of the included studies.

### Data Synthesis

A narrative synthesis of the findings from the included studies will be carried out using descriptive analysis. Our data synthesis will describe the data extracted, including patient demographics, intervention and comparator details, and outcomes. Where possible, statistical analysis will be employed to explore differences in preoperative and postoperative outcomes (clinician-reported outcomes, PROMs, and adverse outcomes) between single-stage and 2-stage facial reanimations in individual studies. The risk of bias and quality of evidence of the included studies will also be discussed in the context of the research question and the wider literature of facial reanimation. Discussion points may be drawn from such analyses. GraphPad Prism (version 10.0.0; GraphPad Software) will be used for any statistical analysis undertaken in the review. The heterogeneity of clinician-reported outcomes and PROMs used in facial palsy indicates that there is limited scope for a high-quality meta-analysis, and thus, a meta-analysis will not be carried out. Subgroup or subset analysis is unlikely to be useful for examining the sufficiently specific original review question.

## Results

This systematic review formally began in February 2025, although it has been planned since July 2024. The final systematic review will be performed and reported according to this protocol and the PRISMA guidelines [37,39]. A PRISMA flow diagram and checklist have been included to summarize the search and selection strategy up to this date (Multimedia Appendices 3 and 4). Initially, the search returned 2120 records, and 56 records are currently being screened for eligibility. Data from the final included studies will be extracted, collated, and analyzed. The risk of bias and the quality of evidence will be ascertained. A narrative synthesis will be used to summarize the results of the systematic review. Where possible, statistical analyses will be presented using graphs and figures. The final systematic review is expected to be published in December 2025.

## Discussion

The authors hypothesize that the included studies, particularly those that are more recent, will show that patients who undergo single-stage facial reanimation using free functional muscle flaps have quicker reinnervation and recovery times than patients who undergo 2-stage facial reanimation. As mentioned, there is an expected heterogeneity in the outcomes used by studies, which may mean that the hypothesis is difficult to objectively display. For example, it is unlikely that the data extracted and analyzed from the included studies will allow for a forest plot that would summarize the results of this review’s outcomes. This systematic review, however, will still be clinically useful as the most up-to-date review comparing the single-stage and 2-stage facial reanimation techniques. The information collated and discussed in this review may be used to aid clinicians and patients in shared decision-making. Moreover, future research questions may be raised, allowing for the conception of further studies on this topic.

To the best of our knowledge, Natghian et al [30] published the only systematic review comparing single-stage and 2-stage facial reanimation using free functional muscle flaps. The study specifically examined smile excursion as an outcome and concluded that single-stage facial reanimation may produce superior smile excursion and symmetry when compared with the 2-stage technique; however, the study lacked data on the spontaneity of smiling after surgery, which is a key indicator of smile quality. Previous research has shown that 2-stage facial reanimation produces a greater spontaneous smile [11]. Since the study by Natghian et al [30], further articles discussing facial reanimation with free functional flaps have been published; however, a contemporary systematic review that discusses the most recent literature is yet to be performed. The proposed systematic review will be a necessary addition to the current literature because it will provide an updated comparison of the single-stage and 2-stage facial reanimation techniques using a broader range of outcomes than that in the study by Natghian et al [30], such as recovery time, nerve regeneration, and smile spontaneity. Therefore, the authors argue that this proposed systematic review’s strengths are its recency and wide scope.

A limitation of the proposed review is the heterogeneity of the outcomes used in the topic, which limits the scope for statistical analyses such as meta-analysis. There are likely to be differences even within the single-stage and 2-stage techniques, such as different free functional muscle flaps and different innervations. While the proposed review will extract these data where possible, the scope for subgroup analysis is likely limited due to the limited number of studies. Many included studies will be prone to selection bias because clinicians may prefer one technique due to psychosocial factors, such as appearance, and clinical characteristics, such as comorbidities, which may impact recovery [58,59]. Therefore, the findings may be undermined. Hence, tools, such as ROBINS-I and GRADE, will be used to provide the risk of bias and quality of evidence of each included study for transparency. To disseminate the results, the authors will aim to present this review at a relevant conference and to publish it in a relevant journal.

## Appendix

Multimedia Appendix 1 Example search strategy for MEDLINE via Ovid.

Multimedia Appendix 2 Example search strategy for ClinicalTrials.gov via Cochrane Library.

Multimedia Appendix 3 Up-to-date PRISMA flow diagram.

Multimedia Appendix 4 Up-to-date PRISMA-P (Preferred Reporting Items for Systematic Review and Meta-Analysis Protocols) checklist.

## Abbreviations

CFNG: cross-facial nerve graft
GRADE: Grading of Recommendations, Assessment, Development, and Evaluations
PICO: population, intervention, comparison, and outcomes
PRISMA: Preferred Reporting Items for Systematic Reviews and Meta-Analyses
PROMs: patient-reported outcome measures
ROBINS-I: Risk of Bias in Nonrandomized Studies-of Interventions
