# Supplementary material for: Single-Stage Versus 2-Stage Facial Reanimation With a Free Functional Muscle Flap: Protocol for a Systematic Review
Source: JMIR Res Protoc. 2025 Aug 21;14:e64009. doi: 10.2196/64009 (PMC12411792; doi:10.2196/64009)
Supplement: Multimedia Appendix 1 [file resprot_v14i1e64009_app1.docx]

**Multimedia Appendix 1.** Example search strategy for MEDLINE via Ovid.

1. Facial Paralysis/
2. Bell Palsy/
3. Facial Nerve Diseases/
4. Smiling/ph [Physiology]

| 1. Facial palsy.ti,ab. |
| --- |

1. Facial palsies.ti,ab.
2. Facial paralyses.ti,ab.
3. Facial Paresis.ti,ab.
4. Facial Pareses.ti,ab.
5. Facial plegia.ti,ab.
6. (Paralys#s adj3 fac$3).ti,ab.
7. (Palsy adj3 fac$3).ti,ab.
8. (palsies adj3 fac$3).ti,ab.
9. (pares#s adj3 fac$3).ti,ab.
10. (plegia adj3 fac$3).ti,ab.
11. 1 or 2 or 3 or 4 or 5 or 6 or 7 or 8 or 9 or 10 or 11 or 12 or 13 or 14 or 15
12. Facial Paralysis/su [Surgery]
13. Bell Palsy/su [Surgery]
14. Surgery, Plastic/
15. Facial Nerve/su [Surgery]
16. Gracilis Muscle/su, tr [Surgery, Transplantation]
17. *Gracilis Muscle/
18. Nerve Transfer/
19. Masseter Muscle/su [Surgery]
20. facial reanimation.mp.
21. Facial animation.mp.
22. (Reanimation adj5 fac$3).ti,ab.
23. (Reanimation adj5 smile).ti,ab.
24. (Reanimation adj6 facial paralys#s).ti,ab.
25. (Reanimation adj6 facial palsy).ti,ab.
26. (Reanimation adj6 facial pares#s).ti,ab.
27. (Surg$ adj6 facial paralys#s).ti,ab.
28. (Surg$ adj6 facial palsy).ti,ab.
29. (Surg$ adj6 facial palsies).ti,ab.
30. (Surg$ adj6 facial pares#s).ti,ab.
31. (Graft adj6 fac$3).ti,ab.
32. (Surg$ adj6 gracilis).ti,ab.
33. (Transfer adj6 gracilis).ti,ab.
34. (Transfer adj6 flap).ti,ab.
35. 17 or 18 or 19 or 20 or 21 or 22 or 23 or 24 or 25 or 26 or 27 or 28 or 29 or 30 or 31 or 32 or 33 or 34 or 35 or 36 or 37 or 38 or 39
36. 1-stage.ti,ab.
37. One stage.ti,ab.
38. Single stage.ti,ab.
39. 1-step.ti,ab.
40. One step.ti,ab.
41. Single step.ti,ab.
42. One procedure.ti,ab.
43. Single procedure.ti,ab.
44. One surgery.ti,ab.
45. Single surgery.ti,ab.
46. 41 or 42 or 43 or 44 or 45 or 46 or 47 or 48 or 49 or 50
47. 16 and 40 and 51
48. 2-stage?.ti,ab.
49. Two stage?.ti,ab.
50. Double stage?.ti,ab.
51. Multi-stage?.ti,ab.
52. 2-step?.ti,ab.
53. Two step?.ti,ab.
54. Double step?.ti,ab.
55. Two procedure?.ti,ab.
56. Double procedure?.ti,ab.
57. Two surg$.ti,ab.
58. Multiple surg$.ti,ab.
59. Multiple procedure?.ti,ab.
60. 53 or 54 or 55 or 56 or 57 or 58 or 59 or 60 or 61 or 62 or 63 or 64
61. 51 or 65
62. 16 and 40 and 66
