# Supplementary material for: Single-Stage Versus 2-Stage Facial Reanimation With a Free Functional Muscle Flap: Protocol for a Systematic Review
Source: JMIR Res Protoc. 2025 Aug 21;14:e64009. doi: 10.2196/64009 (PMC12411792; doi:10.2196/64009)
Supplement: Multimedia Appendix 2 [file resprot_v14i1e64009_app2.docx]

**Multimedia Appendix 2.** Example search strategy for ClinicalTrials.gov via Cochrane Library.

1. MeSH descriptor: [Facial Paralysis] explode all trees
2. MeSH descriptor: [Facial Nerve Diseases] explode all trees
3. MeSH descriptor: [Bell Palsy] explode all trees
4. MeSH descriptor: [Smiling] explode all trees and with qualifier(s): [physiology - PH]
5. Facial pals*:ti,ab
6. Facial paralys?s:ti,ab
7. Facial pares?s:ti,ab
8. Facial plegia:ti,ab
9. Paralys?s NEAR/3 face:ti,ab
10. Paralys?s NEAR/3 facial:ti,ab
11. Pals* NEAR/3 face:ti,ab 6
12. Pals* NEAR/3 facial:ti,ab
13. Pares?s NEAR/3 face:ti,ab
14. Pares?s NEAR/3 facial:ti,ab
15. Pegia NEAR/3 face:ti,ab
16. Plegia NEAR/3 facial:ti,ab
17. #1 or #2 or #3 or #4 or #5 or #6 or #7 or #8 or #9 or #10 or #11 or #12 or #13 or #14 or #15 or #16
18. MeSH descriptor: [Facial Paralysis] explode all trees and with qualifier(s): [surgery - SU]
19. MeSH descriptor: [Bell Palsy] explode all trees and with qualifier(s): [surgery - SU]
20. MeSH descriptor: [Surgery, Plastic] explode all trees
21. MeSH descriptor: [Plastic Surgery Procedures] explode all trees
22. MeSH descriptor: [Facial Nerve] explode all trees and with qualifier(s): [surgery - SU]
23. MeSH descriptor: [Gracilis Muscle] explode all trees and with qualifier(s): [surgery - SU]
24. MeSH descriptor: [Gracilis Muscle] explode all trees and with qualifier(s): [transplantation - TR]
25. MeSH descriptor: [Nerve Transfer] explode all trees
26. MeSH descriptor: [Masseter Muscle] explode all trees and with qualifier(s): [surgery - SU]
27. Facial reanimation:ti,ab
28. Facial animation:ti,ab
29. Reanimation NEAR/5 face:ti,ab
30. Reanimation NEAR/5 facial:ti,ab
31. Animation NEAR/5 facial:ti,ab
32. Reanimation NEAR/5 smile:ti,ab
33. Surg* NEAR/6 face:ti,ab
34. Surg* NEAR/6 facial:ti,ab
35. Surg* NEAR/6 paralys?s:ti,ab
36. Surg* NEAR/6 gracilis:ti,ab
37. Transfer NEAR/6 gracilis:ti,ab
38. Graft NEAR/6 fac*:ti,ab
39. Functional NEAR/6 flap:ti,ab
40. #18 or #19 or #20 or #21 or #22 or #23 or #24 or #25 or #26 or #27 or #28 or #29 or #30 or #31 or #32 or #33 or #34 or #35 or #36 or #37 or #38 or #39
41. #17 and #40
