# Supplementary material for: Single-Stage Versus 2-Stage Facial Reanimation With a Free Functional Muscle Flap: Protocol for a Systematic Review
Source: JMIR Res Protoc. 2025 Aug 21;14:e64009. doi: 10.2196/64009 (PMC12411792; doi:10.2196/64009)
Supplement: Multimedia Appendix 3 [file resprot_v14i1e64009_app3.pdf]

PRISMA 2020 flow diagram for new systematic reviews which included searches of databases and registers only

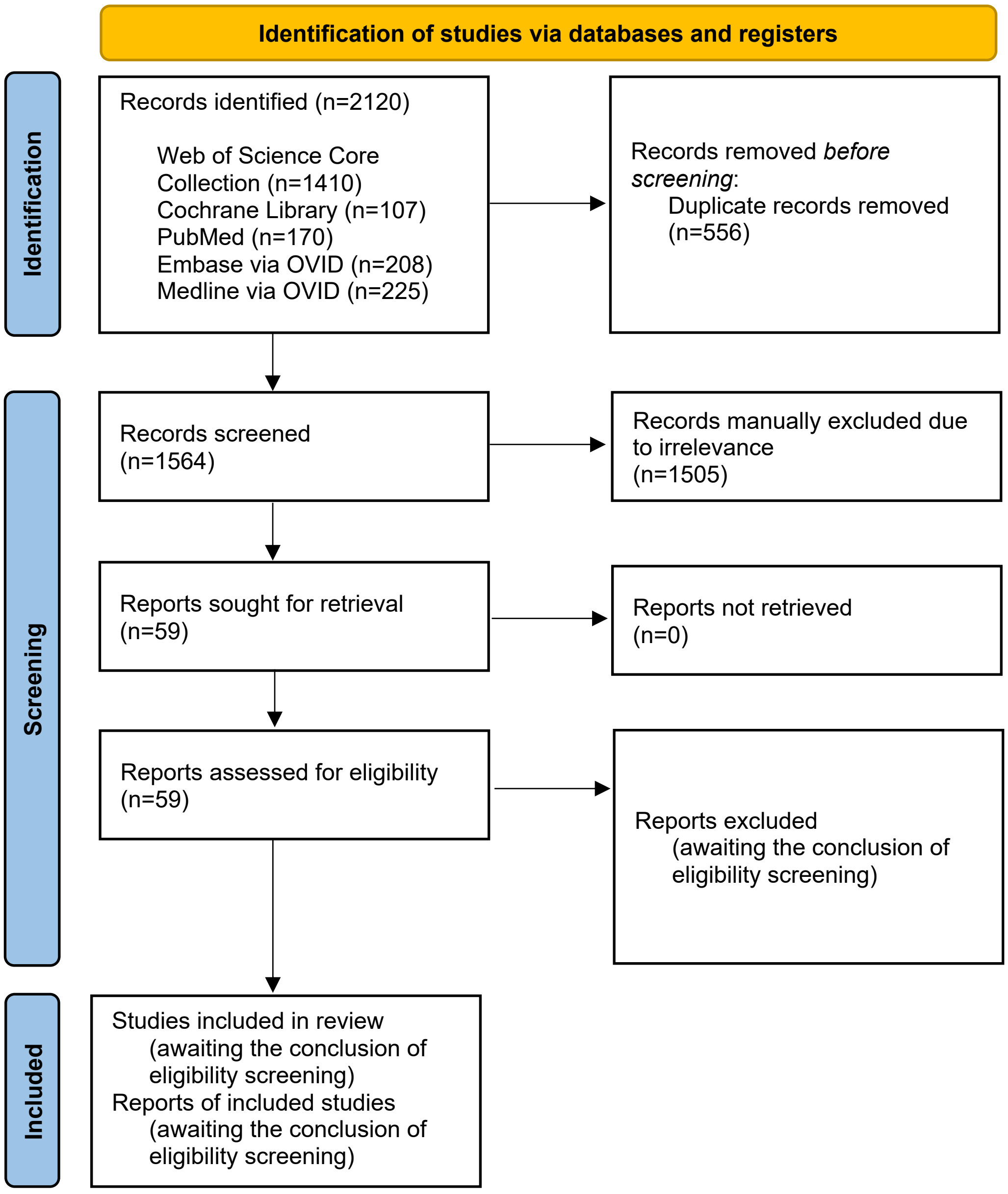

Source: Page MJ, et al. BMJ 2021;372:n71. doi: 10.1136/bmj.n71.

This work is licensed under CC BY 4.0. To view a copy of this license, visit https://creativecommons.org/licenses/by/4.0/
